# Low-volume precision spray for plant pest control using profile variable rate spraying and ultrasonic detection

**DOI:** 10.3389/fpls.2022.1042769

**Published:** 2023-01-10

**Authors:** Yulong Nan, Huichun Zhang, Jiaqiang Zheng, Kunqi Yang, Yufeng Ge

**Affiliations:** ^1^ College of Mechanical and Electronic Engineering, Nanjing Forestry University, Nanjing, China; ^2^ School of Mechanical Engineering, Yancheng Institute of Technology, Yancheng, China; ^3^ Co-Innovation Center of Efficient Processing and Utilization of Forest Resources, Nanjing Forestry University, Nanjing, China; ^4^ Department of Biological Systems Engineering, University of Nebraska-Lincoln, Lincoln, NE, United States

**Keywords:** profile variable rate spraying, ultrasonic sensor, profiling control, precise pesticide application, sprayer

## Abstract

Spraying chemical pesticides is one of the important means to control plant pest, and the profile variable spraying is an important technology to achieve precise pesticide application. A profiling tracking control method and an improved algorithm based on CMAC-PID (Cerebellar Model Articulation Controller- Potential Induced Degradation) were proposed in the paper. The test results of the sprayer profiling tracking of the tree canopies showed that the profiling control system using the improved algorithm had significantly better dynamic tracking performance, and the overall mean tracking error was reduced by 35.0%, compared with the traditional CMAC-PID. A spray flow calculation method based on tree canopy volume and leaf area density was proposed. Outdoor testing of the profile variable spraying and conventional spraying was carried out. There was no significant difference between the two spraying methods in terms of droplet coverage, VMD (Volume Median Diameter), NMD (Number Median Diameter), spray quality parameter and relative span coefficient, as well as droplet deposition density. The spray coefficient of variation was reduced by 25.9% and 21.9% inside and outside the tree canopy, respectively. The mean value of the ground deposition coverage of the profile variable spraying and the traditional spray was 13.0% and 33.2%, respectively, indicating a significant impact on the ground droplet deposition coverage by the two spraying methods. The spray flow rate of the profile variable spraying could be decreased by 32.1% compared to the conventional spraying. Profile variable spraying would reduce the cost associated with pesticide use and environmental pollution.

## 1 Introduction

Plant pest brought huge economic losses to the planting industry, and spraying chemical pesticides was one of the important means to control plant pest. Excessive use of pesticides posed potential risks to the environment, food safety and human health (Pan et al., 2020; Villette et al., 2021). In the prevention and control of traditional agricultural and forestry diseases and insect pests, continuous spraying of pesticides cause pesticide drift and pesticide overuse, causing environmental pollution, and pesticide residues in agricultural and forestry products, as well as soils and waterways (Wen et al., 2019; Dai et al., 2020; Salcedo et al., 2020). Traditional chemical application is known as uniform rate and has been practiced for decades in forest pests and diseases management. It gives no consideration to population density, canopy traits and differences between each individual plant and causes lots of problems, such as large investments, chemical over-prescriptions and pollutions (Zhang et al., 2022). Pesticides for crop disease control have limited future potential, and European countries have initiated green agreements to reduce the use of chemical pesticides for plant disease control (Dietz-Pfeilstetter et al., 2021). Low-volume precision spray was an important way to solve the above problems, but also could achieve the purpose of cleaner production.

Precision forestry means applying the right amount of pesticides in the right place at the right time. If the chemicals could be more efficiently applied, less chemical would be used and the pesticide runoff and leaching would be reduced (Zhang et al., 2017; Ye et al., 2022). The importance of proper selection of spray application methods is being increasingly recognized in precision agricultural and forestry management. With the growing awareness of environmental conservation and public health, pesticide application requires accurate, efficient, scientific, and reasonable operation according to the actual conditions and circumstances (Fang et al., 2021; Li et al., 2021). Effective agricultural and forestry diseases and insect pests control means not only maximizing the effectiveness of the pesticide application, but also minimizing the unintended effects (health hazard and environmental pollution).

The profile variable spraying was one of the main ways to achieve precise pesticide application (Osterman et al., 2013). The profile variable spraying was to adjust the nozzle group to reach the ideal spray distance according to the tree canopy characteristics of agricultural and forestry plants, and change the spray parameters (spray volume and air flow, etc.) in real time to obtain the optimal spray effect (Song et al., 2015; Nan et al., 2019). Ultrasonic (Jordi et al., 2011; Li et al., 2016), LIDAR (Light Detection and Ranging) (Chakraborty et al., 2019; Berk et al., 2020), and imaging (Kise & Zhang, 2008; Mora et al., 2016) were the smart approaches to canopy quantification (Rosell & Sanz, 2012).

The osmanthus tree is a plant of the lignum vitae family and is susceptible to pests and diseases during growth, causing the leaves to wither and fall off. The ultrasonic detection system was used, in real time, to detect the height, width, volume and leaf area index of the tree canopy characteristics (Jeon et al., 2011), estimate the desired application amount, and adjust the spray parameters of the application system (Jeon & Zhu, 2012; Gangadharan et al., 2019). The tree canopy height and width information measured by the ultrasonic sensor was used as the input of a multilayer perceptron neural network to reliably estimate the canopy volume, which was used to change the flow parameters of the spray system, and the pesticide usage was reduced by about 34.5% (Maghsoudi et al., 2015). A profile sprayer based on RGB imaging was developed, whose spraying system adjusted the pesticide spray flow according to the tree canopy image information, and the mean pesticide saving was 23.53% (Hočevar et al., 2010). A sprayer integrated with the Kinect system detected the distance to the tree canopy and the LWA (Leaf Wall Area) density, adjusted the spray flow rate, and improved the efficiency of pesticide spraying (Xiao et al., 2017; Zhang et al., 2019). LiDAR sensor measured the canopy calculation volume in real time, and was applied to variable sprayer to adjust spray flow (Cai et al., 2017; Zeng et al., 2020).

Therefore, a prototype profile sprayer would be developed in this paper, which consisted of three main parts: (a) a description of the prototype sprayer and its electronic system; (b) proposing the profiling control algorithm of the sprayer mechanism and a method to calculation the flow rate; (c) comparing conventional spraying with profile variable spraying to evaluate the performance of profile variable spraying. The purpose of this study was to detect the feature information of the tree canopy through the ultrasonic sensors installed on the sprayer, and to automatically adjust the spray mechanisms to fit the contour of the tree canopy, so as to realize the profile variable spraying. The use of profile variable spraying to achieve on-demand application of pesticides, reasonably reduce the amount of pesticides used, and reduce environmental pollution.

## 2 Materials and methods

### 2.1 Profiling tracking control method

#### 2.1.1 Calculating the tracking profiling target angle of profile sprayer

**Figure 1:**
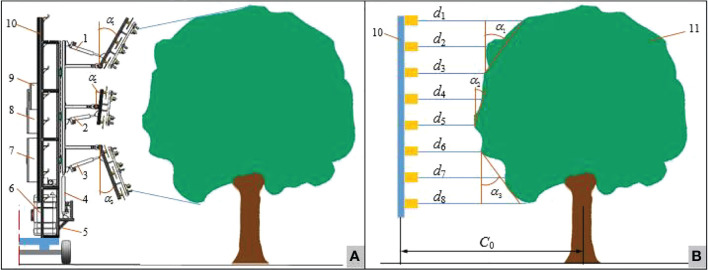
Schematic diagram of single-sided fruit tree profile sprayer profiling and its array ultrasonic detection canopy distance information **(A)** Schematic diagram of single-sided fruit tree profile sprayer profiling **(B)** Schematic diagram of array ultrasound detection of canopy distance information 1. Profiling mechanism module A_up 2. Profiling mechanism module B 3. Profiling mechanism module A_down 4. Lifting slide module 5. Bracket module 6. Liquid supply system 7. Flow and air volume control box 8. Profiling Mechanism profiling control box 9. Canopy phenotyping traits detection control box 10. 8-channel array ultrasonic detection module 11.Fruit tree canopy.

An 8-channel ultrasonic sensors array as the detection module detected the fruit tree canopy, and directly obtained the distances from ultrasonic sensors to the tree canopy, as shown in **Figure 1B**. The ultrasonic sensor array measured the distance between the sensors and the tree canopy and obtained *D* as:


(1) $$D=[d_{1},\;d_{2},\cdots ,d_{7},\;d_{8}\;]$$

In the formula, *d*_1_~*d*_8_ were the distance values obtained by the ultrasonic sensors array along the tree canopy from top to bottom.

The profiling modules A_up, B, and A_down were the sprayer arm modules, and their mounting positions were shown in **Figure 1A**. Equations to calculate profile angles *α*_1_ *、α*_2_ *、α*_3_ , corresponding to the profiling module A_up, B, and A_down, respectively, were:


(2) $$\alpha_{1}=\{\begin{array}{l} \arctan((d_{1}-d_{3})/d_{us})\cdot 180^{\text{o}}/\pi ,\;(d_{1}<C_{0})\;\\ \arctan((d_{2}-d_{3})/d_{us})\cdot 180^{\text{o}}/\pi ,\;(d_{1}>C_{0},d_{2}<C_{0})\\ \alpha_{1}\_L1\;,\;(d_{1},d_{2}>C_{0})\; \end{array}$$

(3) $$\alpha_{2}=\{\begin{array}{l} \mathrm{arctanA}((d_{4}-d_{5})/d_{us})\cdot 180^{\text{o}}/\pi ,\;(d_{4},d_{5}<C_{0})\\ \alpha_{2}\_L1\;,\;(d_{4}>C_{0}) \end{array}$$

(4) $$\alpha_{3}=\{\begin{array}{l} \arctan((d_{8}-d_{6})/d_{us})\cdot 180^{\text{o}}/\pi ,\;(d_{8}<C_{0})\;\\ \arctan((d_{7}-d_{6})/d_{us})\cdot 180^{\text{o}}/\pi ,\;(d_{8}>C_{0},d_{7}<C_{0})\\ \alpha_{3}\_L1\;,\;(d_{7},d_{8}>C_{0})\; \end{array}$$

where, *C*_0_ is the distance from the ultrasonic sensor array on the sprayer to the center of the tree row (m); *d_us_* is the spacing between the ultrasonic sensors (m); *α*_1__*L*1*、α*_2__*L*1*、α*_3__*L*1 was the previous measurement value of the profiling angle *α*_1_ *、α*_2_ *、α*_3_ (°).

Because the shape of the actual tree canopy changed randomly, when the tree canopy was detected by ultrasonic sensors, the profiling angle value of a single calculation varied greatly. This large variation was not conducive to the actual profiling tracking control using the sprayer. Therefore, we used the mean of 4 adjacent angle values as the actual profiling angle value *Pα_i_* of this position, namely:


(5) $$P\alpha_{i}=\frac{\alpha_{i}\_L3+\alpha_{i}\_L2+\alpha_{i}\_L1+\alpha_{i}}{4}(i=1,2,3)$$

where, *α*_*i*__*L*1*、α*_*i*__*L*2*、α*_*i*__*L*3 was the profiling angle of the first 3 measurements, respectively.

The 8-channel ultrasonic sensor array was placed 1m in front of the profile variable sprayer. When the profile sprayer continued to move 1m forward, the actual profiling angle calculation result was extracted as the target angles, which was sent to the profiling control system to drive the three sprayer sets (A_up, B, A_down) to the desired angles.

#### 2.1.2 Electronic circuit system of controlled profile mechanism

**Figure 2:**
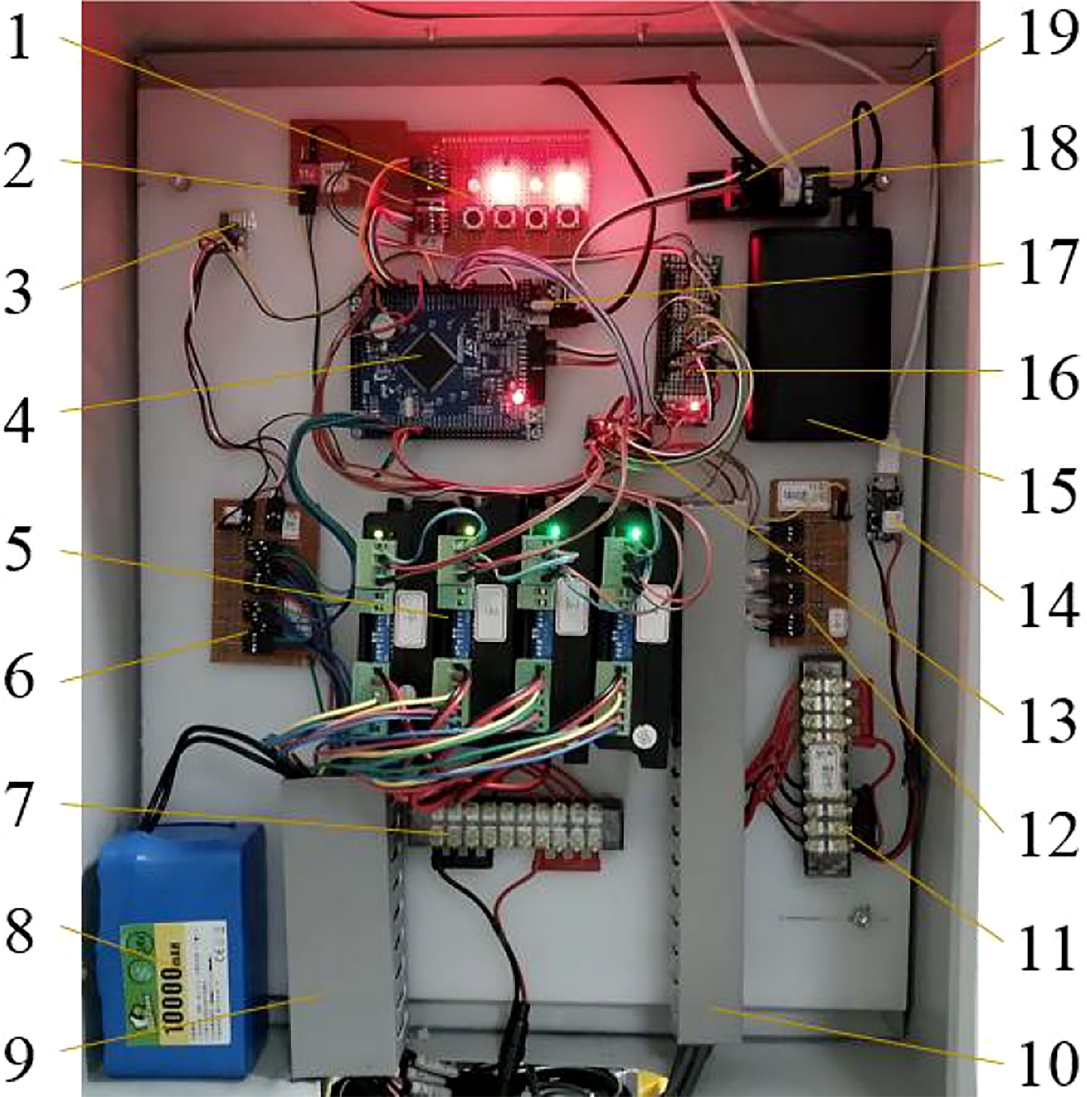
Profiling control circuit system of fruit tree profile variable sprayer 1. Button LED module 2. DIP switch 3.2.4G wireless serial port module 4. STM32F1 control board 5. Stepper motor drivers 6. Pull-up circuit module one 7. Terminal block one 8.24V power supply 9.4-channel stepper motor lines, limit Bit lines and wire slot 10. Encoder wiring and wire slot 11. Terminal block two 12. Pull-up circuit module two 13. Level conversion board 14. Booster module 15. 5V power supply 16. Power adapter board 17. NRF24L01 Module 18. USB extender 19. ST-LINK V2 debugger.

The electronic control system of the profile variable sprayer was shown in **Figure 2**. The GPIOC6~9 ports of the STM32F1 (STM32F103ZET6, STMicroelectronics Group) control board were connected to the pulse ports (Pul-) of the four-way stepper motor driver (DQ-2HD542, Xinghua Oubang Electric Technology Co., Ltd.) through the level conversion module to control the ON/OFF of the four-way stepper. GPIOG2~5 were connected to the direction port (Dir-) of the four-way stepper motor driver through the level conversion module to control the direction of the four stepper push rods.

The 4 encoders were connected to the corresponding IO ports of the STM32F1 control board through the pull-up circuit module two, and respectively detect the real-time rotation angles of the profiling mechanism modules A_up, B and A_down, and the position of the lifting and sliding module, and feedback to STM32F103ZET6 microprocessor. The limit control lines of the four stepping push rods were connected to the corresponding IO ports of the STM32F1 control board through the pull-up circuit module one to ensure the safe operation of the four stepping push rod. The 2.4G wireless serial port module (Si24R1 type, Nanjing Zhongke Microelectronics Co., Ltd.) was connected to the serial port of the STM32F1 control board, which was used to receive the target value of the profiling angle.

#### 2.1.3 The control algorithm improvement of the profiling mechanism control system

The control system of profiling mechanism module A or B adopted the compound control algorithm of CMAC (Cerebellar Model Articulation Controller) neural network and PID (Proportional Integral Derivative), which was as follows (Albus, 1975):


(6) $$u_{n}(k)=\sum_{i=1}^{c}w_{i}a_{i}$$

(7) $$u(k)=u_{n}(k)+u_{p}(k)$$

where, *a_i_* was the binary selection vector, *c* was the CMAC network normalization parameter, *u_n_(k)* was the output of the CMAC network, *u_p_(k)* was the output of the conventional PID controller, and *u(k)* was the calculated output value of the CMAC and PID composite controller. The actual mapping method and adjustment index of CMAC can be found in the literature (Albus, 1975).

For the actual stepping push rod control, when the instantaneous control amount *u(k)* was too large, the stepping motor in the stepping push rod would be blocked. In order to avoid the occurrence of stepper motor blocking, the traditional solution was to limit *u(k)* to a fixed safety threshold. But this would also lead to a decrease in the real-time tracking performance of the system. Therefore, this article proposed a piecewise function *f_seg_* limit *u(k)*:


(8) φ=sign(u(k))

(9) $$t=\{\begin{array}{l} t+1,(\varphi_{0}=\varphi \;\;∥\;\left|u(k)\right|>f_{0})\\ 0\;,(\varphi_{0}\neq \varphi \;∥\;\left|u(k)\right|\leq f_{0}) \end{array}$$

(10) $$\varphi_{0}=\varphi$$

(11) $$f_{seg}=\{\begin{array}{l} f_{0}\;,(t<t_{0})\\ \begin{array}{l} f_{0}+15(t-t_{0})\;,(t_{0}\leq t<t_{1})\\ f_{0}+15(t_{1}-t_{0})+200(t-t_{2})\;,(t_{1}\leq t<t_{2})\\ f_{1}\;,(t\geq t_{2}) \end{array} \end{array}$$

where,*φ* was the direction value controlled by the current stepper motor, *φ*_0_ was the direction value controlled by the previous stepper motor, *sign* was a sign function, and *f_0_* was a fixed safety threshold frequency (Hz). *f_1_* was the upper limit of the control frequency under load (Hz), *t_0_,t_1_* was the start time of the linear increase of the first and second stage frequencies, respectively (ms),*t_2_* was the end time of frequency increase (ms). In this experiment, *f_0 =_* 2500Hz, *f_1 =_* 8500Hz, *t_0 =_* 60ms, *t_1 =_* 80ms, *t_2 =_* 108ms.

Then the actual control value *u(k)* of the stepping putter was:


(12) $$u(k)=\{\begin{array}{l} \lambda \left|u\left(k\right)\right|,\;(u(k)\leq f_{0})\\ \lambda f_{seg}\;,(u(k)>f_{0}) \end{array}$$

### 2.2 Spray flow calculation

**Figure 3:**
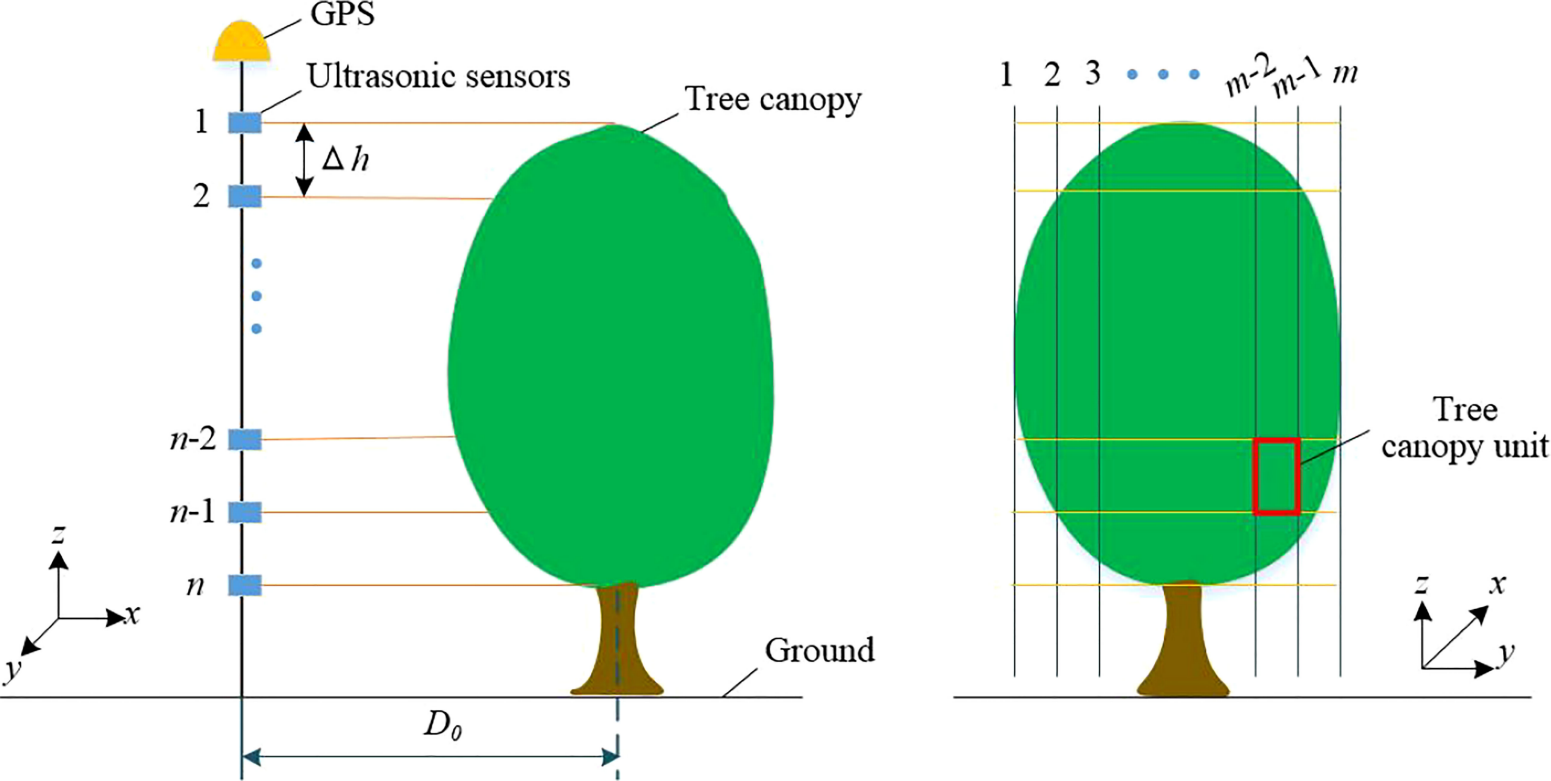
Schematic diagram of ultrasonic sensor detecting tree canopy feature information.


**Figure 3** was a schematic diagram of how the ultrasonic sensors array was used to measure the tree canopy features. The canopy unit volumes *V_nm_* were calculated by the distances between the tree and the ultrasonic sensor array (Zaman & Schumann, 2005):

(13) $$V_{nm}=(D_{0}-d_{nm})\cdot \Delta h\cdot v\cdot \Delta t\;$$

where, *d_nm_* was the ultrasonic measurement distance of the canopy unit at position[*n,m*], (m); Δ*h* was the distance between ultrasonic sensors, (m); *v* was the travel speed of the sprayer (m/s), Δ*t* is the reciprocal of the measuring frequency of the ultrasonic sensors, (s); *D*_0_ is the distance from the sprayer to the center of the tree row, (m).

The leaf area density was estimated by the mean value of ultrasonic echo (Nan et al., 2019):


(14) $$\rho_{snm}=f(\;\rho_{nm},\;y_{nm}\;)$$

where, *ρ_snm_* was the leaf area density of the canopy unit at the position[*n,m*], (g/m^3^); *ρ_nm_* was the density of the canopy unit at the position [*n,m*], (m^2^/m^3^); *y_nm_* was the mean value of ultrasonic echo at the position [*n,m*] of the canopy unit.

The canopy volume parameter was considered when calculating the actual spray application rate. The required pesticide application amount *PV*(L) in the volume of tree canopy unit was:


(15) $$\left\{ \begin{array}{l} k_{LA}=\frac{\rho_{snm}}{S_{\text{max}}}\\ PV=k_{LA}V_{nm}P_{\text{unit}} \end{array}\right.$$

where, *P*_unit_ was the application rate per unit volume, (L/m3); *P*_unit_ =0.1L/m^3^ (Li et al., 2017). *k*_LA_ was the canopy leaf area density coefficient, *S*_max_ was the theoretical maximum leaf area density of the tree canopy, *S*_max_=5.3*m* ^2^ *m* ^−3^ 。

Then, the spray flow rate *Q* (mL/s) was:


(16) Q=PV/Δt

### 2.3 Calculation of spray air volume

For air-driven sprayers, the appropriate air volume could not only improve the penetration and deposition of droplets in the tree canopy, but also reduce the amount of pesticide drift. At present, the calculation method of air volume was mainly based on the principle of replacement and the principle of final velocity. Using *N_b_* independent fans, the air volume of each fan was adjusted according to the volume and density of the tree canopy, so as to realize the adjustment of the air volume of different spray units.

According to the displacement principle, the air volume required by a single spray unit was:


(17) $$q=\frac{(H_{1}+H_{2})\cdot v\cdot D_{0}\cdot k_{m}\cdot k_{s}}{2}$$

where, *H*_1_ was the height of the air outlet (m), *H*_2_ was the height of the spray unit (m), *v* was the speed of the sprayer (m/s), *D*_0_ was the distance from sprayer to center of tree row (m), *k_m_* was the canopy quality factor, *k_s_* was air volume loss factor.


(18) $$H_{2}=N_{z}\cdot \Delta h$$

where, *Δh* was the distance between adjacent ultrasonic sensors, *N_z_* was the number of canopy cells in the z-direction corresponding to a single nozzle spray.


(19) $$k_{m}=\frac{1}{2}+\frac{\rho_{nm}}{\rho_{\max}}$$

where, *ρ*_max_ was the theoretical maximum density value of the tree canopy (m^2^/m^3^).

### 2.4 Evaluation method

**Figure 4:**
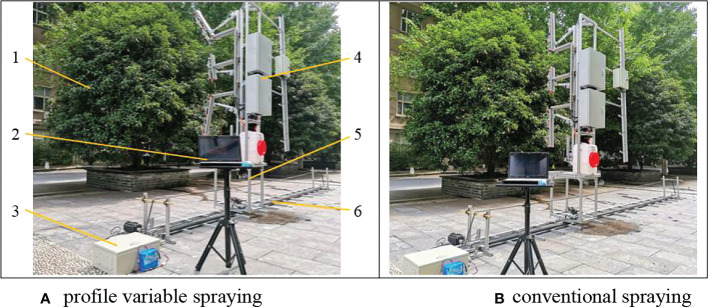
Outdoor spray test scene **(A)** profile variable spraying **(B)** conventional spraying 1.*Osmanthus* tree 2. Computer 3. Ground rail mobile control cabinet 4. Sprayer 5. Mobile rail carrying platform 6. Mobile rail.

**Figure 4** was a field diagram of an outdoor spray test. Six *Osmanthus* trees on the campus of Nanjing Forestry University (geographical location: north Latitude 32°4ʹ52ʺN, Longitude 118°48ʹ37ʺE) were selected and named as tree T1~T6. The profile variable spraying and conventional spraying tests were carried out on trees T1~T6, and the sprayer moving speed was 1m/s. Outdoor temperature was 18°C~30°C, humidity was 50%~60%, wind speed was 0.1~0.3m/s. The distances among trees T1~T6 were 5m. The test site was a flat surface for easy installation of the mobile rails. The sprayer used in the test was mounted on a linear rail, which allowed the speed of the sprayer to be precisely controlled, while also allowing the sprayer to travel parallel to the tree rows. Conventional spraying meant that the sprayer arm was kept vertical and the spray volume and air volume are always kept at a set constant amount. Profiling spraying meant that the spraying machine held the arm to match the canopy profile at all times, and the spray volume and air volume were adjusted to the canopy characteristics. The control algorithm for profiling spraying was more difficult than for conventional spraying, and the cost of using the machine was almost the same.

According to the recently formulated ISO FDIS 22522 standard: “Crop Protection Equipment-Field Measurement of the Spray Distribution of Trees and Shrubs”, the spray tests were carried out following the chemical spraying procedure of the trees. According to the height of the tree, three areas (upper, middle, and lower) were divided, and three areas were divided according to the width of the tree (left, middle, and right). The radial depth was divided into 2 areas (inner layer, outer layer), which formed 18 spray sampling areas. 3 pieces of water-sensitive paper were placed in the center of each sampling area, and after spraying, they were stored in a plastic bag. Therefore, a total of 324 (3 repetitions x 3 heights x 3 widths x 2 depths x 6 trees) spray samples were obtained under each spray configuration condition. In order to evaluate the spray deposition on the ground, the ground where each tree was located was divided into 3 areas (left, middle, and right), and 3 sheets of water sensitive paper were arranged in each area, and the collection width was 3m, that was, the side of each tree was 1.5m.

After each test, the water-sensitive papers were collected, and the Imagepy software (Chongqing Liuliushanxia Plant Protection Technology Co., Ltd.) was used to analyze the image of the water-sensitive paper, and the parameters were extracted from the water-sensitive paper. These parameters include: droplet deposition density, coverage percentage, coefficient of variation (CV), volume median diameter (VMD), number median diameter (NMD), spray quality parameter (*Q_s_*) and relative span factor (Δ).

The calculation method of spray quality parameter (*Q_s_*) was:


(17) $$Q_{s}=VMD/NMD$$

The calculation method of the relative span factor (Δ) was:


(18) $$\Delta =(D_{0.9}-D_{0.1})/VMD$$

where, *D*_0.1_, *D*_0.9_ was droplet diameter than which 10%, 90% of the total volumes of the liquid droplets have smaller diameter, respectively.

One-way ANOVA statistical analysis was used to analyze the significant effect of spraying method (conventional spray *vs*. profiling spray) on different spray parameters. The one-way ANOVA was calculated (citation) as the equations (19-23) and the significance was determined by checking the table according to the F-value.


(19) $$M_{total}=\sum_{i}^{r}\sum_{j}^{n_{r}}X_{ij}$$

(20) $$M_{level}=\sum_{i}^{r}\left(X_{ij}-M_{total}\right)$$

(21) $$SSR=\sum_{j}^{n_{r}}r*(M_{\text{level}j}-M_{\mathrm{total}})$$

(22) $$SSE=\sum_{j}^{n_{r}}\sum_{i}^{r}\left(X_{\mathrm{ij}}-M_{\mathrm{total}}\right)$$

(23) $$F=\frac{SSR/(n_{r}-1)}{SSE/(r*n_{r}-n_{r})}$$

where *X* was the sample data, *n_r_* was the number of levels, *r* was the number of groups, *M_total_* is the overall sample mean, *M_level_* was the water mean, *SSR* is the sum of squared deviations between groups, and *SSE* was the sum of squared deviations within groups.

## 3 Results and analysis

### 3.1 The results of the sprayer profiling tracking tree canopies

**Figure 5:**
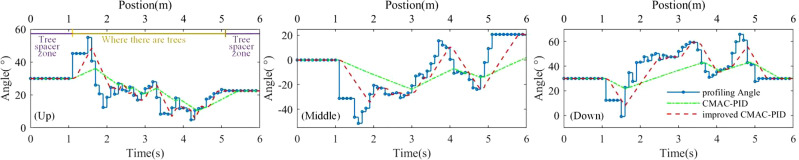
The tracking curves of the sprayer profiling up, middle and down layer of the tree canopy with different control algorithms.

The tracking curves of the sprayer profiling up, middle and down layer of the tree canopy with different control algorithms as shown in **Figure 5**. The sprayer profiling up, middle and down layer of the tree canopy is the parts of the canopy sprayed by the profiling mechanism A_up, B and A_down, respectively. In different layers of the tree canopy, compared with CMAC-PID, the profiling tracking curves of the sprayer using the improved algorithm were closer to the target angle curves, indicating that the profiling control system using the improved algorithm has significantly better dynamic tracking performance. This was because the improved algorithm hierarchically limited the threshold value of the *u*(*k*), and under the premise of preventing blocking, the dynamic speed of the stepping push rod was maximized. Compared with CMAC-PID, when the sprayer profiling control system used the improved algorithm to profile the tree canopy, the mean tracking error was reduced by 33.6%, 34.6%, and 36.7%, respectively, and the overall mean tracking error was reduced by 35.0%.

### 3.2 The results and analysis of outdoor spray

The comparison results of the droplet deposition density obtained with each spray method (profile variable spraying and conventional spraying) are shown in **Table 1**. Compared with the conventional spraying, the droplet deposition density obtained by the profile variable spraying was increased by 44.3% and 69.8% in the inner and outer layers of the tree canopy, respectively. On the whole, the results of one-way analysis of variance showed that the spraying method had a significant effect on the droplet deposition density at the 5% significance level.

**Table 1:** Droplet deposition density data for different tree zones using the profile variable spraying and conventional spraying.

Zone of the tree		Deposits/cm2				
		Profiling variableSpray	Conventionalspray	P *vs*. C	the meanof P *vs*. C	Significance Level
Inner layer	Up	80	50	59.2%	44.3%	*
	Middle	106	70	52.1%		*
	Down	74	61	21.5%		NS
Outer layer	Up	115	55	110.4%	69.8%	*
	Middle	99	73	36.4%		*
	Down	100	62	62.6%		*

*Significant at 5% level; NS, non-significant. P vs. C means that profile variable spraying vs. conventional spraying.

When the profile variable spraying configuration was adopted, the nozzle groups adapted to the shape of the tree canopy contour, the spray distance was reduced, the spray direction was approximately perpendicular to the tangent of the tree canopy contour in the spray area, and the probability of droplets hitting the leaves was significantly increased. This is the main reason for the obvious increase in the mean droplet deposition density in the outer layer areas of the tree canopy.

Meanwhile, when the nozzle groups adapted to the shape of the tree canopy contour, the distance between the air outlet and the tree canopy of the spray area was reduced, and the direction of the air outlet was approximately perpendicular to the tangent of the tree canopy contour of the spray area, which enhanced the ability of the air to penetrate the tree canopy. At this time, the turbulence of the airflow increased the transport capacity of the droplets inside the tree canopy, which was the reason for the obvious increase in the mean droplet deposition density in the inner area of the tree canopy (Hong et al., 2018).

The results of the spray coefficient of variation were shown in **Table 2**. When the sprayer adopted the profile variable spraying method for ultra-low-volume spray, the spray coefficient of variations in different areas of the tree canopy was less than 0.7, which met the standard requirements of NY/T 650-2013 “Sprayer (Apparatus) Operation Quality”. However, when the sprayer adopted the conventional spraying method, only the spray coefficient of variation in the middle zone of the tree canopy in outer layer met the standard requirements. For profile variable spraying method, the spray coefficient of variation in the inner and outer layers zones of the tree canopy was reduced by 25.9% and 21.9%, respectively, indicating that the overall spray uniformity has been significantly improved. The reasons for the above results were as follows. When the sprayer is in the profile variable spraying mode, the sprayer profiled the canopy, and it has the following two advantages: (a) The spray distance was reduced, and the spray direction was approximately perpendicular to the tangent of the canopy contour of the spray area; (b) The distance between the air outlet and the canopy of the spray area was reduced, and the direction of the air outlet was approximately perpendicular to the tangent of the canopy contour of the spray area. With the interaction of the two advantages, the turbulence of the airflow has a stronger ability to disturb the leaves of the tree canopy in the outer and inner layers, and the ability of the airflow to transport the droplets was significantly enhanced in the canopy.

**Table 2:** spray coefficient of variation .

Zone of the tree		Coefficient of Variation			
		Profiling VariableSpray	Conventionalspray	P *vs*. C	the mean ofP *vs*. C
Inner layer	Up	0.472	0.772	-38.8%	-25.9%
	Middle	0.511	0.593	-13.9%	
	Down	0.541	0.722	-25.1%	
Outer layer	Up	0.381	0.829	-54.0%	-21.9%
	Middle	0.438	0.342	28.1%	
	Down	0.433	0.719	-39.7%	

The droplet coverage results of the artificial targets with each spray method (profile variable spraying and conventional spraying) were indicated in **Table 3**. Compared with conventional spraying, the mean value of the droplet coverage obtained by the profile variable spraying increased by 6.8% and 32.8% in the inner and outer layers of the tree canopy, respectively. On the whole the results of the one-way analysis of variance showed that the spray method (profile variable spraying and conventional spraying) had no significant effect on the droplet coverage on the water-sensitive paper. This showed that the profile variable spraying has no obvious irregularities, and the spray performed by the profile variable spraying was acceptable. Taking into account the droplet coverage rate on the tree canopy, **Table 3** showed that the profile variable spraying used less pesticide spray, and overall it could achieve the same or better droplet coverage with conventional spraying.

**Table 3:** Droplet coverage results using the profile variable spraying and conventional spraying.

Zone of the tree		Droplet Coverage/%				
		profile variable spraying	Conventionalspray	P *vs*. C	the mean ofP *vs*. C	Significance Level
Inner layer	Up	0.115	0.11	5.2%	6.8%	NS
	Middle	0.16	0.126	26.3%		*
	Down	0.141	0.159	-11.2%		NS
Outer layer	Up	0.234	0.183	27.9%	32.8%	NS
	Middle	0.323	0.284	13.7%		NS
	Down	0.277	0.176	56.7%		*

*Significant at 5% level; NS, non-significant. P vs. C means that profile variable spraying vs. conventional spraying.

Spray quality results of profile variable spraying, and conventional spraying were indicated in **Table 4**. In each case, the results of the one-way analysis of variance showed that the spray method had no significant effect on VMD, NMD, and spray quality (Qs).

**Table 4:** Spray quality results of profile variable spraying and conventional spraying.

Zone of the tree		Spray quality						
		Profiling Spray			conventional spraying			Significance Level
		VMD	NMD	Qs	VMD	NMD	Qs	VMD/NMD/Qs
Inner layer	Up	193	128	1.49	226	159	1.5	NS
	Middle	219	158	1.42	227	148	1.58	*
	Down	236	154	1.51	244	160	1.6	NS
Outer layer	Up	213	137	1.58	265	173	1.64	NS
	Middle	269	173	1.71	263	168	1.63	NS
	Down	267	166	1.67	278	178	1.74	NS

*Significant at 5% level; NS, non-significant. P vs. C means that profile variable spraying vs. conventional spraying.

Relative span coefficient results of profile variable spraying, and conventional spraying were indicated in **Table 5**. In each case, the results of the one-way analysis of variance showed that the spray mode had no significant effect on *D*_0.1_, *D*_0.9_ and the relative span coefficient (Δ).

**Table 5:** Relative span coefficient results of profile variable spray and traditional spray.

Zone of the tree		Relative span factor						
		Profiling Spray			conventional spraying			Significance Level
		D_0.1_	D_0.9_	Δ	D_0.1_	D_0.9_	Δ	D_0.1_/D_0.9_/Δ
Inner layer	Up	79	374	1.54	109	440	1.47	NS
	Middle	99	400	1.4	96	425	1.47	NS
	Down	89	415	1.39	106	483	1.54	*
Outer layer	Up	83	438	1.79	114	533	1.7	NS
	Middle	117	615	1.84	119	625	1.91	NS
	Down	94	537	1.64	120	550	1.55	NS

* Signiﬁcant at 5% level; NS, non-signiﬁcant. P vs. C means that proﬁle variable spraying vs. conventional spraying.

The droplet coverage results of water-sensitive paper on the ground were shown in **Table 6**. From **Table 6**, it could be seen that the mean droplet coverage on the water-sensitive papers of the conventional spraying at different positions was significantly higher than that of the profile variable spraying. On the whole, the mean droplet coverage on the ground water-sensitive papers of conventional spraying and profile variable spray was 16.5% and 50.9%, respectively. Compared with conventional spraying, there were two main reasons for reducing of droplet coverage on the surface water-sensitive paper using the profile variable spraying. On one hand, profile variable spraying sprayed on demand according to the characteristics of the tree canopy parameters, reducing the spray amount. On the other hand, when the sprayer adopted profile variable spraying, the nozzles were facing the contour of the tree canopy in the spray area, the probability of droplets hitting the canopy was greatly increased, reducing the droplet drift on the ground. The results of one-way analysis of variance showed that the spraying method had a significant impact on the droplet coverage at 3 different locations on the ground.

**Table 6:** Droplet coverage on ground water sensitive paper (3 positions) in profile variable spraying and conventional spraying.

Zone of the ground	Coverage/%			Significance Level
	Profiling Spray	conventional spraying	P *vs*. C(%)	
Left	12.3	35.0	-64.9	*
Middle	12.9	32.5	-60.3	*
Right	13.7	32.2	-57.5	*
Mean	13.0	33.2	-61.0	N/A

*: Significant at 5% level; NS: non-significant. P vs. C means that profile variable spraying vs. conventional spraying.

**Figure 6:**
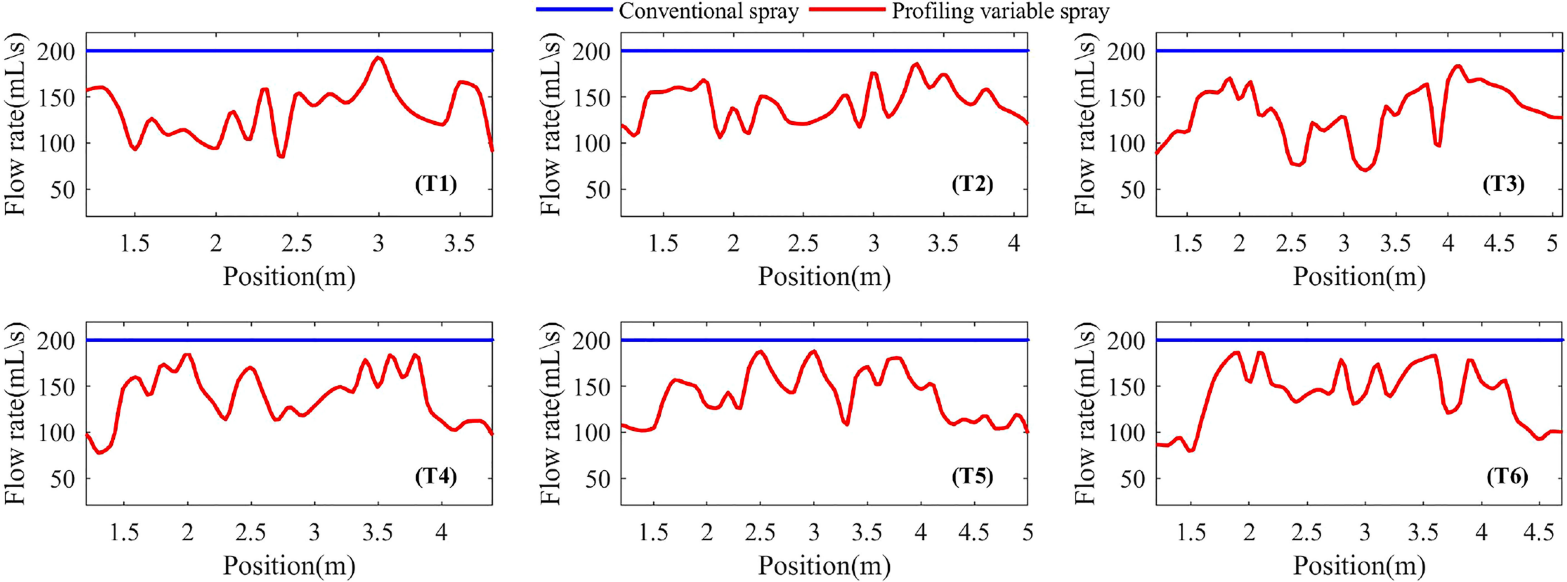
Spray flow rate in different spray methods for Tree T1~T6 canopy.

One of the goals of this study was to estimate the spray saving of the profile variable spraying compared to the conventional spraying. In terms of sustainability, this was one of the most important achievement of the tool: more efficient on canopy and more spray saving. According to the tree canopy information obtained by the ultrasonic sensors detecting the tree canopy, the spray flow rate was calculated to control the duty ratio of the solenoid valve, so that the pesticide could be applied to each tree as needed. **Figure 6** showed the spray flow rate of the sprayer to the tree T1~6 canopies, respectively using the profile variable spraying and the conventional spraying.

In **Figure 6**, the blue horizontal line was the conventional spraying flow rate, the red curve was the profile variable spraying flow rate change rate, and the difference between the blue horizontal line and the red curve represents the saved spray volume. The important thing was that the profile variable spraying could better arrange the spray flow according to the canopy volume and leaf area density information. Compared with conventional spraying, the sprayer used profile variable spraying to spray trees T1~T6, and the spray flow was saved by 33.7%, 29.5%, 35.0%, 32.2%, 31.2% and 30.9%, respectively, and the mean spray flow was saved by 32.1%.

## 4 Discussion

A more promising way proves to be variable rate spraying based chemical application technology that fully utilizes spatial technology. The key idea of variable rate spraying is “applying the right amount of chemical in the right place at the right time”. In forestry settings, however, there are some facts which make variable rate spraying system less preferred. The main reason is lack of methods to quickly measure trees properties. The pesticide saving based on canopy phenotypic characteristics was not only related to canopy morphology, but also related to complex factors. This meant that the profiling spray should comprehensively consider the canopy morphology, canopy density, tree species, volume and other factors, so as to achieve the purpose of effectively saving pesticides. The sprayer arms were manually adjusted to match the canopy shape, and pesticide spray flow was changed in real time based on the canopy shape captured by RGB camera, with 23% savings of pesticides (Hočevar et al., 2010). Therefore, the percentage of pesticide savings in this study has obvious advantage. At the same time, when the amount of pesticides used was significantly reduced, there was no significant difference in pesticide spray deposition and distribution, which could ensure good pest control. In the variable application of the vineyard, the pesticide saving rate reached 58% (Gil, Escolà, Rosell, Planas, & Val, 2007), which was better than that in this study. This was due to the difference in the morphology of the tree species. Using a retrievable sprayer to apply pesticides in the vineyard, the pesticide recovery rate was 16.28%, which could achieve pesticide savings and reduce pesticide drift, and give a new way to save pesticides (Shenzhong et al., 2021).

The effect of accurate application still needs to be verified in production practice, more works are needed to be done to extend the system for practical applications and more points, such as how to install the system on a carrier (for instance a sprayer) and how to test the control effect under different running speeds, should be taken into account. However, as the system running speed increased, some problems may exist which affected the system’s stability and reliability. The existing accurate pesticide application test platforms and spray control systems were different, but these studies could bring inspiration to future research. Real-time positioning algorithm for orchard sprayer was proposed, in order to automatically adjusted the sprayer arms to match the canopy shape and expected the same or better drift reduction, but no spray test verification was carried out (Osterman, Godeša, Hočevar, Širok, & Stopar, 2013). However, array ultrasonic sensors was used to obtain the tree canopy shape in this paper, and the sprayer arms were automatically adjusted to match the tree canopy shape, and a spray test was carried out to verify the spray effects. In future research, the existing profile variable sprayer would be installed on the driving car, and spraying tests of trees in a row would be carried out to further verify the spray performance of the profile variable sprayer. In addition, the method of accurate application based on multi-sensor fusion to extract the comprehensive phenotypic characteristics of trees will be the focus of our further research.

## 5 Conclusions

Presently the infestation of agricultural and forestry diseases and insect pests causes severe problems while the chemical treatment, as the major solution, is of very low efficiency. So, developing precise chemical application methods based on profile characteristics of the tree canopy is urgent and necessary, which bears the promises of low cost and high efficiency of chemical application in diseases and insects treatment and moreover, will protect the ecological environment, shield the operators from the dangerous concoction. This study investigated the use of prototype profile sprayer to realize smart chemical application. Compared with CMAC-PID, the profiling tracking curves of the sprayer using the improved algorithm were closer to the target angle curves, indicating that the profiling control system using the improved algorithm has significantly better dynamic tracking performance, and the overall mean tracking error was reduced by 35.0%.

There was no significant difference between the profile variable spraying and the traditional spray in the spray indicators such as droplet deposition density, droplet coverage, VMD, NMD, spray quality Qs, D_0.1_, D_0.9_, and relative span coefficient Δ, but there was significant difference in droplet density. The spray coefficient of variation was reduced by 25.9% and 21.9% in the inner and outer tree canopy respectively. The mean value of the ground deposition coverage of the profile variable spraying and the traditional spray was 13.0% and 33.2%, respectively. The spray method has a significant impact on the ground droplet deposition coverage. The spray flow rate of the profile variable spraying can be saved by 32.1% on average than that of conventional spraying.

## Data availability statement

The original contributions presented in the study are included in the article/supplementary material. Further inquiries can be directed to the corresponding author.

## Author contributions

Research idea and manuscript writing: YN, HZ and JZ; data analysis: YN, HZ, and KY; experiments: KY and YN; funding acquisition and resources: HZ and JZ; writing-review and editing: YN, HZ, YG and JZ. All authors contributed to the article and approved the submitted version.

## Funding

This work is supported by National Natural Science Foundation of China (NSFC 32171790), Jiangsu Province Modern Agricultural Machinery Equipment and Technology Demonstration Promotion Project (NJ2020-18), Funding for school-level research projects of Yancheng Institute of Technology (xjr2021012), Six Talent Peaks Project in Jiangsu Province (Grant No. NY-058), Qinglan Project Foundation of Jiangsu Province (Grant No. 20161520193) and 333 Project of Jiangsu Province (Grant No. 20186).

## Conflict of interest

The authors declare that the research was conducted in the absence of any commercial or financial relationships that could be construed as a potential conflict of interest.

## Publisher’s note

All claims expressed in this article are solely those of the authors and do not necessarily represent those of their affiliated organizations, or those of the publisher, the editors and the reviewers. Any product that may be evaluated in this article, or claim that may be made by its manufacturer, is not guaranteed or endorsed by the publisher.

## References

[B1] Albus J. S. (1975). Data storage in the cerebellar model articulation controller (cmac). Transaction Asme J. Dynamical Syst. Measurement Controls 97 (3):228–233. doi: 10.1115/1.3426923

[B2] Berk P. Stajnko D. Belsak A. Hocevar M. (2020). Digital evaluation of leaf area of an individual tree canopy in the apple orchard using the lidar measurement system. Comput. Electron. Agric. 169, 105158. doi: 10.1016/j.compag.2019.105158

[B3] Cai J. Wang X. Song J. Wang S. Yang S. Zhao C. (2017). Z development of real-time laser-scanning system to detect tree canopy characteristics for variable-rate pesticide application. Int. J. OF Agric. AND Biol. Eng. 10 (6), 155–163. doi: 10.25165/j.ijabe.20171006.3140

[B4] Chakraborty M. Khot L. R. Sankaran S. Jacoby P. W. (2019). Evaluation of mobile 3d light detection and ranging based canopy mapping system for tree fruit crops. Comput. Electron. Agric. 158, 284–293. doi: 10.1016/j.compag.2019.02.012

[B5] Dai X. Xu Y. Zheng J. Ma L. Song H. (2020). Comparison of image-based methods for determining the inline mixing uniformity of pesticides in direct nozzle injection systems. Biosyst. Eng. 190, 157–175. doi: 10.1016/j.biosystemseng.2019.12.007

[B6] Dietz-Pfeilstetter A. Mendelsohn M. Gathmann A. Klinkenbuß D. (2021). Considerations and regulatory approaches in the usa and in the eu for dsrna-based externally applied pesticides for plant protection. Front. Plant Sci. 12. doi: 10.3389/fpls.2021.682387 PMC8232971 34177998

[B7] Fang S. Ru Y. Liu Y. Hu C. Chen X. Liu B. (2021). Route planning of helicopters spraying operations in multiple forest areas. Forests 12 (12), 1658. doi: 10.3390/f12121658

[B8] Gangadharan S. Burks T. F. Schueller J. K. (2019). A comparison of approaches for citrus canopy profile generation using ultrasonic and leddar® sensors. Comput. Electron. Agric. 156, 71–83. doi: 10.1016/j.compag.2018.10.041

[B9] Hočevar M. Širok B. Jejčič V. Godeša T. Lešnika M. Stajnko D. (2010). Design and testing of an automated system for targeted spraying in orchards. J. Plant Dis. Prot. 117 (2), 71–79. doi: 10.1007/BF03356338

[B10] Hong S. Zhao L. Zhu H. (2018). Cfd simulation of pesticide spray from air-assisted sprayers in an apple orchard: tree deposition and off-target losses. Atmospheric Environ. 175, 109–119. doi: 10.1016/j.atmosenv.2017.12.001

[B11] Jeon H. Y. Zhu H. (2012). Development of a variable-rate sprayer for nursery liner applications. Trans. ASABE 55 (1), 303–312. doi: 10.13031/2013.41240

[B12] Jeon H. Y. Zhu H. Derksen R. C. Ozkan H. E. Krause C. R. Fox R. D. (2011). Performance evaluation of a newly developed variable-rate sprayer for nursery liner applications. Trans. ASABE 54 (6), 1997–2007. doi: 10.13031/2013.40648

[B13] Jordi L. Emilio G. Jordi L. Alexandre E. (2011). Ultrasonic and lidar sensors for electronic canopy characterization in vineyards: advances to improve pesticide application methods. Sensors 11 (2), 2177. doi: 10.3390/s110202177 22319405 PMC3274039

[B14] Kise M. Zhang Q. (2008). Development of a stereovision sensing system for 3d crop row structure mapping and tractor guidance. Biosyst. Eng. 101 (2), 191–198. doi: 10.1016/j.biosystemseng.2008.08.001

[B15] Li L. He X. Song J. Wang X. Jia X. Liu C. (2017). Design and experiment of automatic profiling orchard sprayer based on variable air volume and flow rate. Nongye Gongcheng Xuebao/Transactions Chin. Soc. Agric. Eng. 33 (1), 70–76. doi: 10.11975/j.issn.1002-6819.2017.01.009

[B16] Li Q. J. Yuan P. C. Lin Y. S. Tong Y. K. Liu X. (2021). Pointwise classification of mobile laser scanning point clouds of urban scenes using raw data. J. Applied Remote Sensing. 15 (2). doi: 10.1117/1.JRS.15.024523

[B17] Li H. Zhai C. Weckler P. Wang N. Yang S. Zhang B. (2016). A canopy density model for planar orchard target detection based on ultrasonic sensors. Sensors 17 (1), 31. doi: 10.3390/s17010031 28029132 PMC5298604

[B18] Maghsoudi H. Minaei S. Ghobadian B. Masoudi H. (2015). Ultrasonic sensing of pistachio canopy for low-volume precision spraying. Comput. Electron. Agric. 112 (SI), 149–160. doi: 10.1016/j.compag.2014.12.015

[B19] Mora M. Avila F. Carrasco-Benavides M. Maldonado G. Olguín-Cáceres J. Fuentes S. (2016). Automated computation of leaf area index from fruit trees using improved image processing algorithms applied to canopy cover digital photograpies. Comput. Electron. Agric. 123, 195–202. doi: 10.1016/j.compag.2016.02.011

[B20] Nan Y. Zhang H. Zheng J. Bian L. Li Y. Yang Y.. (2019). Estimating leaf area density of osmanthus trees using ultrasonic sensing. Biosyst. Eng. 186, 60–70. doi: 10.1016/j.biosystemseng.2019.06.020

[B21] Osterman A. Godeša T. Hočevar M. Širok B. Stopar M. (2013). Real-time positioning algorithm for variable-geometry air-assisted orchard sprayer. Comput. Electron. Agric. 98 (Supplement C), 175–182. doi: 10.1016/j.compag.2013.08.013

[B22] Pan D. He M. Kong F. (2020). Risk attitude, risk perception, and farmers' pesticide application behavior in china: a moderation and mediation model. J. Cleaner Production 276, 124241. doi: 10.1016/j.jclepro.2020.124241

[B23] Rosell J. R. Sanz R. (2012). A review of methods and applications of the geometric characterization of tree crops in agricultural activities. Comput. Electron. Agric. 81 (4), 124–141. doi: 10.1016/j.compag.2011.09.007

[B24] Salcedo R. Zhu H. Zhang Z. Wei Z. Chen L. Ozkan E.. (2020). Foliar deposition and coverage on young apple trees with pwm-controlled spray systems. Comput. Electron. Agric. 178, 105794. doi: 10.1016/j.compag.2020.105794

[B25] Shenzhong D. Wei Q. Changjie H. Jie G. Good P. (2021). Experimental study on the liquid recovery system of vineyard sprayer. J. Agric. Mechanization Res. 43 (09), 180–185. doi: 10.13427/j.cnki.njyi.2021.09.033

[B26] Song Y. Sun H. Li M. Zhang Q. (2015). Technology application of smart spray in agriculture: a review. Intelligent Automation Soft Computing 21 (3), 319–333. doi: 10.1080/10798587.2015.1015781

[B27] Villette S. Maillot T. Guillemin J. P. Douzals J. P. (2021). Simulation-aided study of herbicide patch spraying: influence of spraying features and weed spatial distributions. Comput. Electron. Agric. 182, 105981. doi: 10.1016/j.compag.2020.105981

[B28] Wen S. Han J. Ning Z. Lan Y. Yin X. Zhang J.. (2019). Numerical analysis and validation of spray distributions disturbed by quad-rotor drone wake at different flight speeds. Comput. Electron. Agric. 166, 105036. doi: 10.1016/j.compag.2019.105036

[B29] Xiao K. Ma Y. Gao G. (2017). An intelligent precision orchard pesticide spray technique based on the depth-of-field extraction algorithm. Comput. Electron. Agric. 133 (Supplement C), 30–36. doi: 10.1016/j.compag.2016.12.002

[B30] Ye Z. Guo Q. Wei J. Zhang J. Zhang H. Bian L.. (2022). Recognition of terminal buds of densely-planted chinese fir seedlings using improved yolov5 by integrating attention mechanism. Front. Plant Sci. 13. doi: 10.3389/fpls.2022.991929 PMC9589298 36299793

[B31] Zaman Q. Schumann A. W. (2005). Performance of an ultrasonic tree volume measurement system in commercial citrus groves. Precis. Agric. 6 (5), 467–480. doi: 10.1007/s11119-005-4243-x

[B32] Zeng L. Feng J. He L. (2020). Semantic segmentation of sparse 3d point cloud based on geometrical features for trellis-structured apple orchard. Biosyst. Eng. 196, 46–55. doi: 10.1016/j.biosystemseng.2020.05.015

[B33] Zhang X. Fu L. Karkee M. D. Whiting M. Zhang Q. (2019). Canopy segmentation using resnet for mechanical harvesting of apples. IFAC-PapersOnLine 52 (30), 300–305. doi: 10.1016/j.ifacol.2019.12.550

[B34] Zhang H. Zheng J. Zhou H. DORR G. J. (2017). Droplet deposition distribution and off-target drift during pesticide spraying operation. Trans. Chin. Soc. Agric. Machinery 48 (8), 114–122. doi: 10.6041/j.issn.1000-1298.2017.08.012

[B35] Zhang C. Zhou H. Xu L. Ru Y. Ju H. Chen Q. (2022). Wind tunnel study of the changes in drag and morphology of three fruit tree species during air-assisted spraying. Biosyst. Eng. 218, 153–162. doi: 10.1016/j.biosystemseng.2022.04.003

